# First Report on *Bacillus*‐Mediated Gonad Growth and Coloration in Purple Sea Urchin (*Strongylocentrotus purpuratus*) Using a Plant‐Meal Diet

**DOI:** 10.1155/anu/7403059

**Published:** 2026-05-30

**Authors:** Alfonso Rodríguez, Jeremie Bauer, Manuel Acosta, Carmen Paniagua-Chávez, Jorge Olmos

**Affiliations:** ^1^ Marine Biotechnology Department, Center for Scientific Research and Higher Education of Ensenada, Ensenada, Baja California, Mexico, cicese.edu.mx; ^2^ Aquaculture Department, Center for Scientific Research and Higher Education of Ensenada, Ensenada, Baja California, Mexico, cicese.edu.mx

**Keywords:** gonad enhancement, microbial enzymes, probiotics, sea urchin ranching, soybean meal, sustainable feeds

## Abstract

Sea urchin ranching has been proposed as a potential restorative aquaculture activity to manage sea urchins from barrens affecting kelp forests globally. However, the development of sustainable and economically viable formulated feeds for sea urchin ranching is needed. This study evaluates the effect of a plant‐based diet and the incorporation of *Bacillus* probiotics on gonad growth, survival, and gonad coloration in purple sea urchin (*Strongylocentrotus purpuratus*). Over 8 weeks, four dietary treatments were assessed: plant‐based diet without *Bacillus* (AFC) and three probiotic‐supplemented diets (AFSp0, AFSp1, and AFSp2) incorporating 2 × 10^9^ CFU kg^−1^ of *B. subtilis*, *B. velezensis*, and *B. amyloliquefaciens*, respectively. Probiotic treatments achieved mean (±SE) gonadosomatic index (GSI) of 15.30 ± 0.76, 16.10 ± 0.65, and 14.30 ± 0.60 for AFSp0, AFSp1, and AFSp2, respectively. In contrast, *S. purpuratus* in AFC obtained 8.23% ± 0.58% (*p*  < 0.05). Gonad weight increase (GWI) reached 634.10% ± 32.10% in AFSp2 versus 150.00% ± 15.90% in AFC (*p*  < 0.001). Survival rates were higher in probiotic treatments (AFSp0: 81.81% ± 7.87%; AFSp1: 84.84% ± 1.51%; and AFSp2: 92.4% ± 1.51%) compared to AFC (45% ± 2.62%). Low survival in AFC may be related to nondigestible antinutritional factors (ANFs) present in plant ingredients. Finally, spectrophotometric and high‐performance liquid chromatography (HPLC) analyses suggest the presence of putative 9‐cis‐echinenone (9CEC) at high concentrations in probiotic treatments. 9CEC concentration was 13.65 μg/g, 20.40 μg/g, and 17.10 μg/g for AFSp0, AFSp1, and AFSp2, respectively, representing increases of 31.88%, 97.10%, and 65.22% relative to the AFC control treatment (10.35 μg/g). These findings suggest that *Bacillus* strains improve gonad growth, survival, and gonad coloration in *S. purpuratus* fed a plant‐meal diet, potentially through ANFs degradation, nutrients absorption, and carotenoid metabolism enhancement. This research aims to provide practical insights for improving sea urchin ranching while assessing the broader potential of probiotic‐enhanced plant‐based nutrition in sustainable aquaculture systems.

## 1. Introduction

Sea urchin gonads, also known as *uni* or *roe*, represent a high‐value delicacy, with the highest quality *uni* reaching up to USD 1000/kg [1, 2]. High‐quality gonads are characterized by a high gonadosomatic index (GSI > 12%), sweet taste, and bright‐yellow/orange coloration [3–7]. Red sea urchin (*Mesocentrotus franciscanus*) fisheries have traditionally provided commercial *uni* along the Northeastern Pacific, relying on macroalgal forests as a food source [4, 8–11]. However, in the last decade, a combination of marine heatwaves and purple sea urchin (*Strongylocentrotus purpuratus*) overpopulation has devastated kelp forests up to 95% in some regions, resulting in the formation of urchin barrens—low biodiversity areas dominated by herbivores [12–15]. These conditions hinder *M. franciscanus* gonad development and make fishery‐based supply unfeasible. Furthermore, while *S. purpuratus* populations in these barrens represent a potential aquaculture resource, commercial gonad production from these organisms necessitates sustainable and economically viable feeding alternatives.

Gonad enhancement of wild‐caught sea urchins—known as sea urchin ranching—represents a potential short‐term alternative for gonad production [16–18]. Additionally, it has been proposed as a restorative aquaculture strategy to generate economic benefits for coastal communities and induce the production of sustainable food, while controlling urchin overpopulation affecting kelp forests [19–21]. Nonetheless, traditional approaches for urchin ranching use macroalgae‐ and fishmeal‐based formulated diets with synthetic carotenoid supplementation [18, 22–27]. Nonetheless, fishmeal and macroalgae seem unsustainable for developing formulated feed due to declining wild populations, negative effects in marine food webs, and higher production costs [13, 28, 29]. In that sense, alternatives for developing sustainable and economically viable aquafeed for sea urchin ranching need to be explored.

Terrestrial plant meals—like soy—offer viable alternatives as they present both ecological and economic benefits while maintaining adequate nutrient profiles [30]. However, plant meals contain antinutritional factors (ANFs) that impair nutrient digestibility and the survival of aquaculture organisms [31–33]. For example, soy protein accessibility is naturally constrained by complex oligosaccharides (raffinose, stachyose, and verbascose) that contain α‐1,6 and α‐1,2 glycosidic bonds [34–37]. While serving as energy and protein storage mechanisms, these structures limit nutrient bioavailability through detrimental effects on intestinal function (i.e., enteritis) for animals lacking specialized carbohydrase enzymes [37–40]. Hence, using plant‐based formulated feeds for urchin ranching could affect gonad growth, survival, and gonad coloration.

Gonad coloration is key for successful sea urchin ranching (Takagi et al., 2020). Adequate gonad coloration depends on β‐carotene uptake, transformation to 9‐cis‐echinenone (9CEC) in the gut, and transportation to gonads via low‐density lipoproteins (LDLs) [26, 41–45]. Interestingly, soybean and other plant meals naturally contain carotenoids along with carbohydrate, protein, and lipid profiles potentially attractive for sea urchin gonad growth and coloration [28]. However, ANFs in plant meals could compromise gonad growth and sea urchin survival. In that sense, strategies to mitigate these antinutritional properties while preserving gonad growth and coloration are urgently needed.

*Bacillus* probiotics are a promising solution for using plant meals in formulated feeds [ 30, 38, 46, 47]. In particular, *B. subtilis* [48], *B. velezensis* [33], and *B. amyloliquefaciens* [49] are GRAS‐certified probiotics with demonstrated capacity to eliminate ANFs and facilitate soy inclusion in aquafeeds [31, 32, 38]. These *Bacillus* strains have rendered beneficial effects in commercially valuable species for aquaculture [33, 38, 48–52]. Nevertheless, to our knowledge, despite established probiotic benefits, specific application of *Bacillus* in plant‐based formulated feeds for gonad growth and coloration in sea urchins remains unexplored.

The present study addresses this knowledge gap by evaluating the effects of a plant‐based diet with soybean meal supplemented with different *Bacillus* strains on gonad growth, survival, and gonad coloration of *S. purpuratus* obtained from urchin barrens. This research aims to provide practical insights for improving sea urchin ranching while assessing the broader potential of probiotic‐enhanced plant‐based nutrition in sustainable aquaculture systems.

## 2. Materials and Methods

### 2.1. Feed Preparation

The plant‐based feed formula is a noncommercial proprietary recipe, and ingredient composition cannot be provided. Diet preparation followed four key principles: (I) exclusion of fishmeal and fish oil, (II) exclusion of all macroalgae meals, (III) inclusion of terrestrial plant meals, and (IV) inclusion of probiotics.

Dry ingredients were mixed for 15 min (KitchenAid–K5SSWH), 2 × 10^9^ CFU kg^−1^ of *Bacillus* strains Sp0, Sp1, and Sp2 were incorporated with warm water, and mixture was extruded to produce wet pellets (*d* = 1/8″) with the aid of a thermal jacket (Applikon–ZC813H110BW). Pellets were dried at 60°C for 4 h and stored in sealed plastic containers at room temperature until use.

Diets AFSp0, AFSp1, and AFSp2 with *Bacillus*, and AFC control without *Bacillus* contain 18.78% protein, 3.20% lipids, and 55.06% carbohydrates. Formulation utilized terrestrial plant meals as primary sources of macronutrients supplemented with essential vitamins and minerals (Table 1).

**Table 1:** Theoretical proximal composition of feed.

Component	AFC	AFSp0–AFSp2
Macronutrient composition (g kg^−1^)		
Protein	187.8	187.8
Carbohydrates	550.6	550.6
Lipids	32.0	32.0
Fiber	65.4	65.4
Moisture	71.6	71.6
Primary ingredients (g kg^−1^)		
Soybean meal^1^	160	160
Vegetable meals^2^	664	664
Vitamin and mineral premix^3^	5	5
* Bacillus* strains (Sp0, Sp1, Sp2)	0	2 × 10^9^ CFU kg^−1^

^1^Soyarin.

^2^De la Rosa.

^3^Brovel S.A. de C.V.

### 2.2. *Bacillus* Strains Identification and Inclusion

Three *Bacillus* strains (Sp0, Sp1, and Sp2) were obtained from our culture collection at the Center for Scientific Research and Higher Education of Ensenada (CICESE; Ensenada, Baja California, Mexico). Phylogenetic identification and growth conditions of strains were described by Macias et al. [49] and Mercado et al. [33]. Sp0, Sp1, and Sp2 were previously identified as *B. subtilis*, *B. velezensis*, and *B. amyloliquefaciens*, respectively. Strains were cultured at 37°C and 250 rpm in Shaeffer medium until the *T*_4_ sporulation phase was reached and kept at 4°C until use.

*Bacillus* suspension in the stationary phase was applied via atomization onto the ingredients mixture prior to extrusion and drying. Experimental diets were supplied with 2 × 10^9^ CFU kg^−1^ of *Bacillus* Sp0, Sp1, and Sp2 strains, based on previous aquafeed production [31, 33, 49]. *Bacillus* CFU were verified in a Petri dish with LB medium.

### 2.3. Collection, Transport, and Acclimatization of Sea Urchins

Purple sea urchins *S. purpuratus* (*n* = 200) with a mean (±SD) test diameter of 50.06 ± 10.76 mm and wet weight of 55.68 ± 2.98 g were collected from urchin barrens on February 19th, 2024, at Arbolitos, Ensenada, Baja California, Mexico (31°42′48.7″ N 116°42′15.4″ W). Sea urchins were transported to the Marine Biotechnology Department at CICESE in seawater‐filled coolers equipped with portable aeration pumps (JHL‐1201).

Organisms underwent a 1‐week acclimatization period in a 2500 L glass fiber tank without feeding to standardize initial nutritional conditions. A random sample (*n* = 15) of sea urchins was sacrificed to determine the initial GSI, coloration, and established baseline values for experimental comparisons.

### 2.4. Experimental Feeding Conditions

The study evaluated four dietary treatments, three probiotics (AFSp0, AFSp1, and AFSp2) and a control without probiotics (AFC) in a randomized factorial tank design with three replicate units per treatment. The feeding experiment was conducted over 8 weeks from February 26th to April 16th, 2024. Experimental units consisted of 200 L tanks with 22 sea urchins and circulating seawater with a daily exchange equivalent to 30% of the total volume. A natural photoperiod was maintained according to spring conditions (~14:10 light:dark) without artificial illumination.

Sea urchins were randomly distributed among experimental units and fed at 0.8% [3] of their body wet weight every 2 days. The organisms were hand‐fed to ensure equal food access and availability. Tanks were siphoned before feeding to remove fecal matter and uneaten feed. Dead or moribund organisms were removed upon observation but were not replaced.

Water quality parameters were maintained at optimal conditions for *S. purpuratus* cultivation [18, 53]. The seawater temperature was maintained at 17 ± 1°C with a water chiller (Aqualogic DS‐5). Dissolved oxygen (> 6 mg L^−1^) and pH (8.0–8.2) were measured with a Hach multimeter (HQ40D) and salinity (33–35‰) with a RES refractometer (10ATC) three times per week before feeding.

### 2.5. Gonad Extraction

Gonad extraction was conducted based on the method of James et al. [54]. First, sea urchins’ wet weight was measured. Then, coelomic cavities were drained, and organisms were weighed again to record drained body weight. Finally, the gonads were removed using a spoon, thoroughly rinsed with seawater, and weighed using an electronic balance (0.01 g; Weigh Gram TOP‐200).

### 2.6. Gonad Growth and Urchins’ Survival

Biometric parameters were measured at days 0 and 56. After gonad extraction, the following equations were used to evaluate gonad growth parameters [18, 22, 24]:

GSI, %:

(1) GSI=TGWTDW×100.

Weekly gonad index increase (WGII, %):

(2) WGII=GSIf−GSIod×7.

Gonad index increase (GII, %):

(3) GII=GSIf−GSIoGSIo×100.

Gonad weight increase (GWI, %):

(4) GWI=TGWf−TGWoTGWo×100,

where TGW = total gonad weight, TGWf = final gonad weight, TGWo = initial gonad weight, TDW = total drained weight, GSIf = final GSI, GSIo = initial GSI, *d* = days of experiment.

### 2.7. Gonad Color Assessment

Gonad color evaluation followed a scale based on Pearce et al. [22]. A four‐point grading system was employed: Grade 1 (A^+^) = orange; Grade 2 (A) = bright yellow; Grade 3 (B) = pale yellow; and Grade 4 (C) = brown or any other color.

### 2.8. Carotenoids Spectrophotometric Characterization and Quantification

Pooled (*n* = 4 per treatment) gonad samples were treated for carotenoid extraction following an established protocol for reversed‐phase high‐performance liquid chromatography (RP‐HPLC) analysis [42, 55–57]. Briefly, gonads were rinsed with seawater and pooled in 15 mL tubes covered with aluminum foil. Gonads were homogenized in 100% cold acetone (1:2 w v^−1^) and incubated for 15 min. Samples were centrifuged at 8000 × *g* for 5 min and supernatants were recovered; the process was repeated until gonads were no longer colored.

Supernatants were saponified with 10% potassium hydroxide in methanol (1:1 v v^−1^) overnight. After saponification, hexane (1:1 v v^−1^) and 10% sodium chloride solution were added, and the mixture was incubated for 15 min. Aliquots of 1 mL were transferred to an amber microcentrifuge for evaporation under vacuum (Eppendorf–5301) at room temperature, and extracts stored at −20°C. Aliquots were reconstituted in 1 mL of acetone for UV‐VIS spectrophotometric and HPLC analysis. UV‐VIS analysis of gonad extracts was conducted using a Hach D500 spectrophotometer. Absorption spectra were recorded by performing wavelength scans across the 350–550 nm range. Carotenoids concentration was calculated using the molar extinction coefficient reported for echinenone extracted in acetone [58]:

(5) CM=Aε ,

where *C* = molar concentration of echinenone, *A* = maximum absorbance, and *ε* = 119,000 L mol^−1^ cm^−1^. Final echinenone concentration was expressed as µg g^−1^ of wet weight.

### 2.9. HPLC Characterization and Quantification of Carotenoids

Carotenoids characterization was performed through RP‐HPLC analysis using gonad extracts from each treatment. Carotenoid extraction and analysis were performed as mentioned above and based on previously described methods for sea urchins [42, 56–58].

A standard solution of 5 mg of β‐carotene (Sigma–Aldrich Química, >95% purity) in 1 mL of hexane was prepared. A 100 μL aliquot was transferred to an amber microcentrifuge tube for solvent evaporation and reconstituted at the same volume with cold acetone. Characterization was performed by HPLC in an Agilent 1100 equipment with a C18 Phenosphere column (5 μm, 150 mm × 4.6 mm) using an isocratic method (MeOH 100%; 0.5 mL min^−1^; Column T° = 28°C). Detection was performed at 450, 460, and 470 nm (UV‐VIS Gilson 156). The intensity (mAU) measured for the standard solution was scaled by a factor of 0.15 for comparative visualization.

### 2.10. Statistical Analysis

Tanks were used as experimental units (*n* = 3 per treatment) for all analyses. Individual measurements within tanks were averaged to generate tank‐level values prior to statistical testing. Data normality and variance homogeneity were assessed using the Shapiro–Wilk and Levene’s tests, respectively. Nonparametric analyses were employed since normality assumptions were violated for gonad growth parameters. Treatment effects were evaluated using Kruskal–Wallis tests and Dunn’s post hoc tests with Bonferroni correction for multiple comparisons. All statistical analyses were performed using R software (Version 4.3.0, RRID:SCR_000432) with the significance set at *α* = 0.05. Statistics and data visualization were performed using ggplot2, FSA, and dunn.test packages. Graphical representations, figures, and tables were created using R software and BioRender (Toronto, Canada, RRID:SCR_018361).

## 3. Results

### 3.1. Gonad Growth and Urchins’ Survival

**Figure 1:**
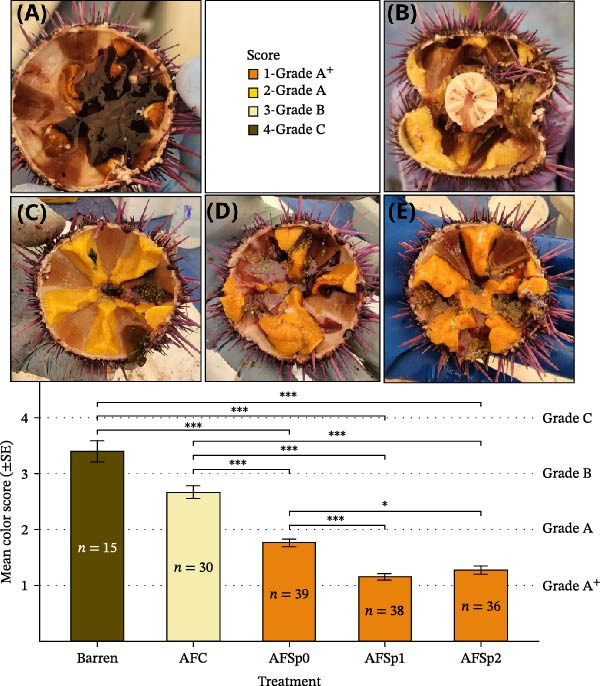
Comparative gonad coloration of *Strongylocentrotus purpuratus* from barrens and experimental treatments. Wild specimens from barren (A) exhibit poor coloration quality (Grade C, brown). Experimental specimens show progressive improvement: control treatment AFC (B) achieved moderate coloration (Grade B, pale yellow), while *Bacillus*‐supplemented treatments AFSp0 (C), AFSp2 (D), and AFSp1 (E) produced superior coloration (Grades A and A^+^). The bar chart shows mean color score (±SE) per treatment group, where lower scores indicate better gonad coloration. Dashed horizontal lines denote grade thresholds (1 = Grade A^+^; 2 = Grade A; 3 = Grade B; 4 = Grade C). Sample sizes (*n*) are indicated within each bar. AFC = control feed without probiotics; AFSp0 = probiotic feed with strain Sp0; AFSp1 = probiotic feed with strain Sp1; and AFSp2 = probiotic feed with strain Sp2;  ^∗∗∗^ *p*  < 0.001;  ^∗^ *p*  < 0.05.

Sea urchins collected from barrens (Figure 1A), exhibited a GSI mean (±SE) value of 2.78% ± 0.52% at the beginning of the experiment and after 8 weeks of treatment Dunn’s post hoc test revealed significant differences between AFC and all *Bacillus* treatments (*H* = 39.00, *df* = 3, *p*  < 0.001; Table 2). No differences were recorded among *Bacillus* treatments (AFSp0 vs. AFSp1 *Z* = −1.13, *p* = 0.686; AFSp0 vs. AFSp2 *Z* = 1.20, *p* = 0.781; AFSp2 vs. AFSp1 *Z* = 2.35, *p* = 0.056).

**Table 2:** Gonad growth and survival metrics for *S. purpuratus* fed the probiotic‐supplemented treatments (AFSp0, AFSp1, and AFSp2) and the control treatment without probiotic (AFC).

Mean (±SE) gonad growth and survival^1^					
Feed	GSI (%)	WGII (%)	GWI (%)	GII (%)	Survival (%)
AFC	8.23 ± 0.58^a^	0.70 ± 0.09^a^	149 ± 15.9^a^	206.93 ± 16.67^a^	45.45 ± 2.62^a^
AFSp0	15.30 ± 0.76^b^	1.58 ± 0.11^b^	564 ± 35.9^b^	464.74 ± 35.24^b^	81.81 ± 7.87^b^
AFSp1	14.30 ± 0.60^b^	1.45 ± 0.10^b^	563 ± 36.0^b^	407.27 ± 2.31^b^	84.84 ± 1.51^b^
AFSp2	16.10 ± 0.65^b^	1.68 ± 0.09^b^	634 ± 32.1^b^	506.74 ± 17.68^b^	92.41 ± 1.51^b^

*Note:* Statistically significant differences are denoted by different superscript letters. AFC = control feed without probiotics, AFSp0 = probiotic feed with strain Sp0, AFSp1 = probiotic feed with strain Sp1, and AFSp2 = probiotic feed with strain Sp2.

^1^The table shows final gonadosomatic index (GSI, %), weekly GSI increase (WGII, %), gonad weight increase (GWI, %), gonadosomatic index increase (GII, %) and survival for sea urchins under every treatment.

Mean (±SE) gonad growth and survival metrics in probiotic treatments differed from the AFC control (Table 2). WGII in probiotic treatments were 1.58% ± 0.11% for AFSp0, 1.45% ± 0.01% for AFSp1 and 1.68% ± 0.09% for AFSp2 (Table 2), which significantly exceeded AFC value of 0.70% ± 0.09% (*H* = 45.62, *df* = 3, *p*  < 0.001). GWI in probiotic treatments were 564.8% ± 35.9% for AFSp0, 563.8% ± 36% for AFSp1 and 634.1% ± 32.1% for AFSp2, differing from the AFC that reached 149.5% ± 15.9% (*H* = 49.39, *df* = 3, *p*  < 0.001, Table 2). GII in probiotic treatments were 465% ± 35.2% for AFSp0, 407% ± 2.31% for AFSp1 and 507% ± 17.7% for AFSp2, which significantly exceeded AFC value of 207% ± 16.7% (*H* = 39.00, *df* = 3, *p*  < 0.001). Survival rates varied amongst treatments; *Bacillus* inclusion treatments achieved higher survival rates (AFSp0 81.81% ± 7.87%, AFSp1 84.84% ± 1.51% and AFSp2 92.4% ± 1.51%) than the AFC control treatment (45.45% ± 2.62%; *H* = 7.97, *df* = 3, *p* = 0.047).

### 3.2. Gonad Color Assessment

**Figure 2:**
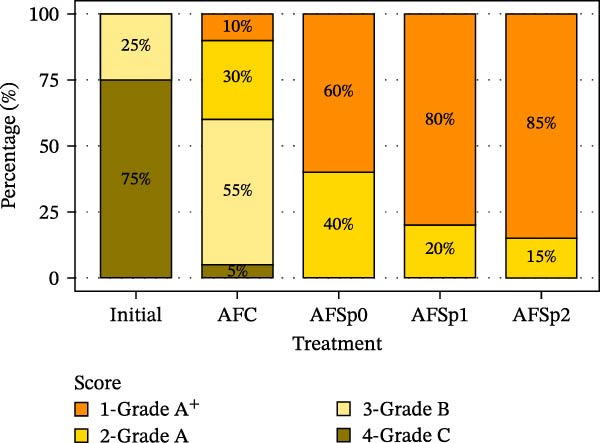
Distribution of gonadal coloration grades in *S. purpuratus* from barren habitat (initial) and following 90‐day feeding trials (*n* = 4 per treatment). Probiotic treatments AFSp0, AFSp1, and AFSp2 produced predominantly Grade A^+^ gonads (60%, 80%, and 85%, respectively), while control treatment AFC yielded primarily Grade A and B gonads (30% and 55%, respectively). Initial barren specimens exhibited poor coloration (75% Grade C). Grade A^+^ (bright orange); Grade A (bright yellow); Grade B (pale yellow); Grade C (brown). AFC = control feed without probiotics; AFSp0 = probiotic feed with strain Sp0; AFSp1 = probiotic feed with strain Sp1; and AFSp2 = probiotic feed with strain Sp2.

Gonad coloration differed between *Bacillus* treatments (Figure 1C,D) and the control (Figure 1B). Sea urchins from barren passed from brown color (Grade 4) to mostly pale‐yellow gonads (Grade 3) in AFC control treatment after 8 weeks (Figure 1A,B). Probiotic treatments achieved ~60/40% in AFSp0 (Grade 1/2), 80/20% in AFSp1 (Grade 1/2), and 85/15% in AFSp2 (Grade 1/2) bright yellow/orange gonad colors (Figure 2).

### 3.3. Carotenoids Spectrophotometric Characterization and Quantification

**Figure 3:**
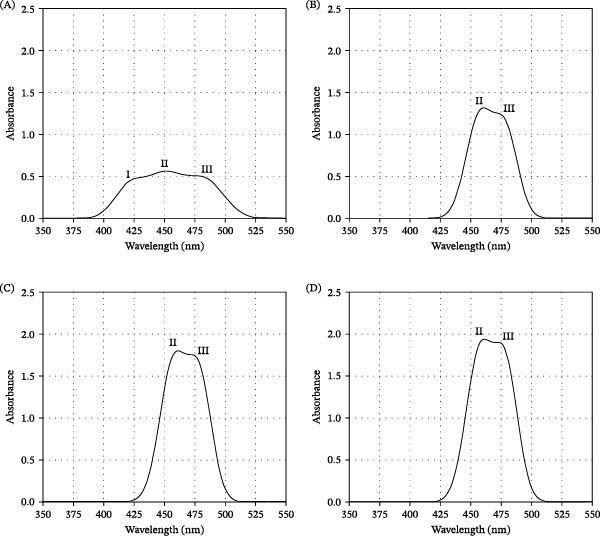
Spectrophotometric analysis of carotenoid composition in *Strongylocentrotus purpuratus* gonads across experimental treatments. AFC (A) displays low spectral intensity (absorbance: 0.563, integral: 50.019). Probiotic treatments (B–D) AFSp0, AFSp1, and AFSp2 exhibit spectral profiles consistent with echinenone. AFSp2 achieved maximum absorbance (D; 1.937, integral: 196.20), followed by AFSp1 (C: 1.799, integral: 175.37) and AFSp0 (B: 1.314, integral: 141.48). AFC = control feed without probiotics; AFSp0 = probiotic feed with strain Sp0; AFSp1 = probiotic feed with strain Sp1; and AFSp2 = probiotic feed with strain Sp2.

*Bacillus* treatments AFSp0 (Figure 3B), AFSp1 (Figure 3C), and AFSp2 (Figure 3D) displayed a spectral profile with a maximum absorption at 457 nm (peak II) and one additional shoulder (III), like that reported for 9CEC in acetone [55, 59]. AFC control treatment showed a different spectral pattern, indicating the presence of another pigment or isomer (Figure 3A). Furthermore, AFC contained the lowest pigment concentration (10.35 μg g^−1^), while probiotic treatments produced 13.65 μg g^−1^ (AFSp0), 17.10 μg g^−1^ (AFSp1), and 20.40 μg g^−1^ (AFSp2), which correspond to 32%, 65%, and 97% more pigment concentration than AFC.

### 3.4. Carotenoids HPLC Characterization and Quantification

**Figure 4:**
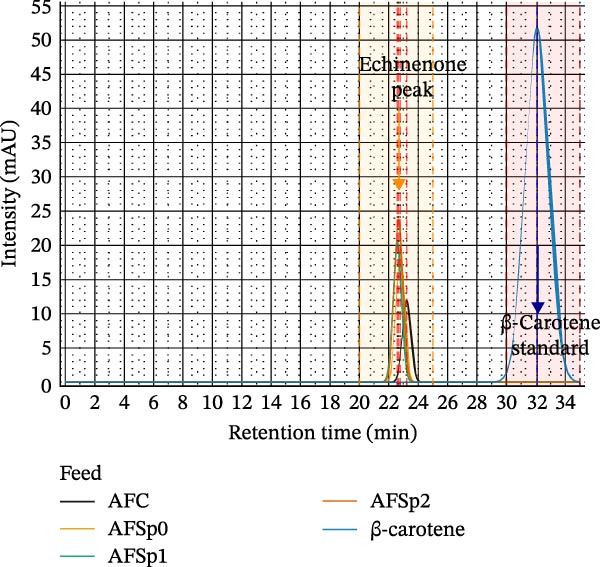
Chromatogram overlay showing probiotic‐mediated carotenoid enhancement in *Strongylocentrotus purpuratus* gonads. HPLC analysis (450 nm detection) of gonad extracts comparing AFC (gray) and probiotic treatments AFSp0 (yellow), AFSp1 (green), and AFSp2 (orange) with β‐carotene reference standard (blue). Orange shading indicates echinenone region (20–25 min); blue shading indicates β‐carotene region (30–35 min). Major peaks were reconstructed using Gaussian modeling (resolution: 0.01 min). β‐carotene standard was scaled by factor 0.15 for comparative visualization (original intensity: 344.99 mAU). AFSp0, AFSp1, and AFSp2 peaks cluster at 22–23 retention time (minutes). AFSp2 shows optimal enhancement (2.0‐fold increase vs. AFC). mAU = micro absorbance units; min = minutes; AFC = control feed without probiotics; AFSp0 = probiotic feed with strain Sp0; AFSp1 = probiotic feed with strain Sp1; and AFSp2 = probiotic feed with strain Sp2.

HPLC chromatograms obtained in this work show a consistent and predominant peak around 22 min of retention time in *Bacillus* treatments (Figure 4). The peak size varied between treatments, with AFSp2 producing the highest, followed by AFSp1 and AFSp0. AFC treatment produced a peak with a slight difference that could correspond to putative all‐trans‐echinenone (ATEC) [42, 60]. AFC recorded a minimal peak height compared with probiotic treatments. The β‐carotene standard exhibited a peak with a higher retention time (Figure 4), as was expected by its more hydrophobic properties [61].

## 4. Discussion

This study represents the first evaluation of gonad growth, survival, and gonad coloration in *S. purpuratus* using a plant‐based formulated feed supplemented with three *Bacillus* probiotic strains. Results indicate that probiotic treatments improved gonad growth, survival, and gonad coloration compared to the control without probiotics. These results suggest that *Bacillus* probiotics are key in developing plant‐based formulated feeds for sea urchin ranching while proposing a viable alternative to traditional macroalgae‐ and fishmeal‐based diets.

*Bacillus* strains employed in this study produce extracellular carbohydrate‐degrading enzymes, including α‐glucosidases, α‐galactosidases, and β‐1,4‐endoglucanases, which facilitate the degradation of starch, soy oligosaccharides, and cellulosic matrices, respectively [33, 49, 62]. Additionally, these strains may convert hydrophobic soy proteins into bioavailable hydrophilic peptides [33]. Moreover, semisolid‐state fermentation has shown complete degradation of glycinin and β‐conglycinin with Sp1 and Sp2 strains, achieving >95% reduction in allergenic protein bands [32, 33]. In that sense, these enzymatic capacities may transform nutrients into readily assimilable metabolites that support *S. purpuratus* gonad growth and survival. This is supported by our results showing that probiotic treatments AFSp0, AFSp1, and AFSp2 achieved superior gonad growth performance compared to the AFC control. Notably, all probiotic treatments surpassed commercial GSI thresholds (>12%), while AFC (8.23 ± 0.58) remained within the minimal commercial viability (6%–12% GSI) established for *S. purpuratus* [4].

**Figure 5:**
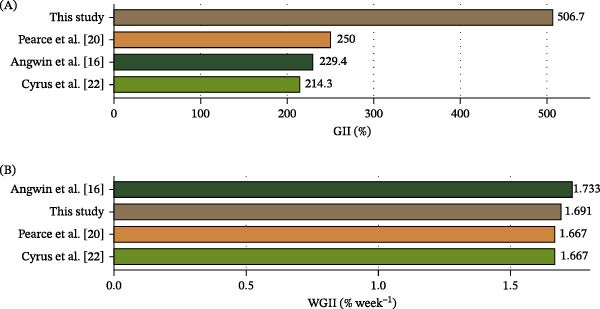
Comparison of gonadal growth metric parameters across sea urchin feeding studies. (A) Gonadal index increase (GII, %) represents the total percentage increase from initial to final measurements. (B) Weekly gonadal index increase (WGII, % week^−1^) represents the percentage increase rate per week. Colors represent the major dietary components used in each study: this study (earthy brown) = soybean meal + *Bacillus* sp. with *Strongylocentrotus purpuratus*; [18] (dark sea green) = kelp‐based diet with *S. purpuratus*; [24] (olive green) = *Ulva* spp. (20%) + maize + soybean protein concentrate with *Tripneustes gratilla;* [22] (sandy brown) = *Ascophyllum nodosum* + *Palmaria palmata* + soybean meal with *Strongylocentrotus droebachiensis*. Values on bars indicate the exact measurements for each parameter.

Our plant‐based diet supplemented with *Bacillus* probiotics matched the WGII performance of macroalgae‐fish‐based diets without probiotic supplementation [18, 22, 24]. We found that AFSp2 treatment achieved WGII values consistent with those studies. However, the GII in AFSp2 was two‐fold greater than values obtained in those previous works (Figure 5), suggesting that *Bacillus* strains may facilitate plant nutrient absorption and ANFs degradation. For example, Mercado et al. [33] demonstrated that *Bacillus* strains Sp1 and Sp2 used in this work possess ANFs degradation capacity when applied to diets with high levels of soy‐based protein. In that sense, when fed a plant‐based diet, *Bacillus* probiotics could have beneficial effects not only on *S. purpuratus’* gonad growth but also survival.

**Figure 6:**
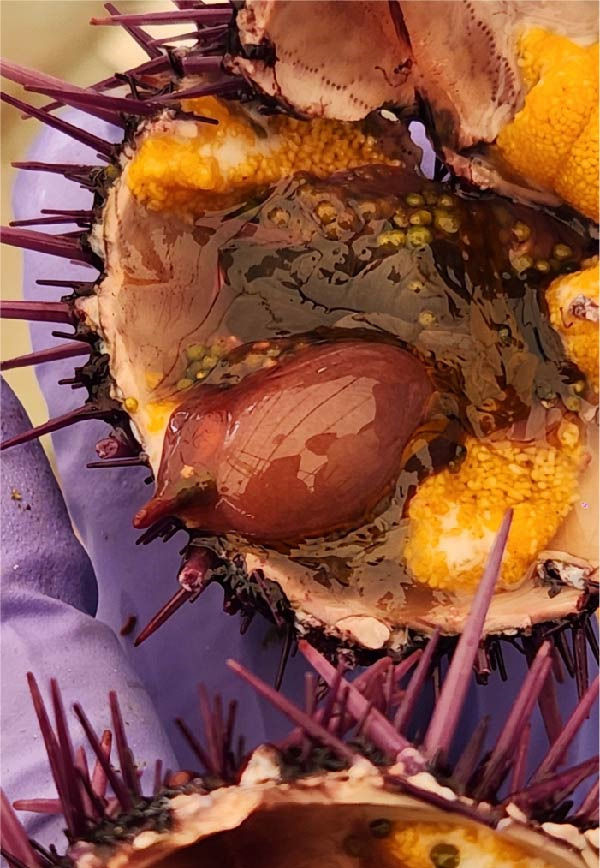
Intestinal tissue in *Strongylocentrotus purpuratus* fed the control treatment (AFC) without probiotic supplementation. Specimens exhibit signs of enteritis, including inflammation.

*Bacillus* species used in this work have been shown to modulate the intestinal microbiome of aquatic species from pathogen‐susceptible toward pathogen‐resistant profiles [31–33, 63, 64], including protection of *Totoaba macdonaldi* and *Oreochromis niloticus* against *Vibrio* and *Aeromonas hydrophila* challenges [33, 49]. In *S. purpuratus*, this protective effect likely operates through two complementary mechanisms: (1) antimicrobial peptide production [49, 65–67] and (2) ANFs degradation [33]. The latter being particularly relevant here given that AFC control specimens exhibited marked intestinal inflammation (Figure 6). This inflammation is consistent with prolonged exposure to allergenic proteins and nondigested oligosaccharides [35, 39, 68–70], and may have affected survival in AFC.

The low survival recorded in AFC (45.45% ± 2.62%) could be related to nondigested ANFs present in the plant‐based control diet without probiotics rather than water quality issues. This interpretation is supported by the progressive mortality pattern: AFC specimens exhibited spine loss beginning in weeks 2–3, reduced locomotion and feeding response, and visible intestinal inflammation—a progression consistent with soy‐induced pathology previously documented [31, 37, 70]. In contrast, in probiotic treatments, sea urchins had a high survival and maintained intestinal integrity, suggesting that enzymatic ANFs degradation may not only improve nutrient accessibility but also prevents the inflammatory damage associated with these compounds [32]. Additionally, water quality parameters showed no significant differences between treatments, remaining within optimal ranges for *S. purpuratus* throughout the experiment [18, 53].

Previous studies on sea urchin pigment evaluation have identified 9CEC as the predominant carotenoid peak in HPLC analysis [42, 56, 71]. High‐quality gonad coloration (bright yellow/orange) depends on efficient β‐carotene oxidation to 9CEC in the urchin gut by specific microbial species [42, 58, 72], followed by LDL‐mediated transport and accumulation [44, 45, 73]. In this study, spectrophotometric and HPLC analyses of carotenoid composition in urchins treated with *Bacillus* suggest that putative 9CEC is the most abundant pigment in bright‐yellow and orange gonads. This is supported by the gonad coloration obtained in plant‐based treatments with and without *Bacillus*, suggesting that the plant‐based diet may contain sufficient bioactive compounds to support putative 9CEC production. However, *Bacillus* strains appear critical for inducing high‐quality gonad coloration in *S. purpuratus* under these experimental conditions. *Bacillus* treatments produced spectral patterns with predominant absorption peaks at 457 and 482 nm, aligning with 9CEC behavior in acetone [55, 59]. Therefore, the bright yellow/orange gonads obtained in *Bacillus* treatments may correlate with the predominant presence of putative 9CEC in their acetone extracts.

Conversely, the AFC control treatment without *Bacillus* produced pale yellow gonads with a distinct spectral pattern in acetone, suggesting the presence of an alternative pigment or isomer. ATEC is the second most common isomer found in sea urchin gonads, and its presence is associated with less bright or pale‐yellow coloration [43]. Thus, the pale‐yellow gonads and distinct spectral pattern found in the AFC control suggest the presence of putative ATEC in this treatment.

HPLC analysis revealed a predominant peak in all *Bacillus* treatments that could be attributed to putative 9CEC, and a slightly different peak in the AFC control treatment that could correspond to putative ATEC. Tsushima and Matsuno [60] reported slight differences in retention time between these two isomers that match the results found in this work. Moreover, Brewster et al. [43] mentioned that ATEC may be part of the pigment profile found in sea urchin gonads, and its presence is related to the pale‐yellow coloration. Moreover, our HPLC results are in agreement with findings reported in *Strongylocentrotus intermedius* [71] and in *Strongylocentrotus droebachiensis* [74], where echinenone was identified as responsible for the yellowish‐orange gonad coloration.

Therefore, gonad coloration, chromatographic characterization, and HPLC quantification all suggest the predominant presence of putative 9CEC in *Bacillus* treatments and putative ATEC in the AFC control. *Bacillus* treatments produced the highest pigment concentrations and the best quality coloration. In this context, the highest putative 9CEC concentrations correspond to orange‐colored gonads, bright‐yellow gonads correspond to moderate putative 9CEC levels, and pale‐yellow coloration corresponds to the putative ATEC presence.

## 5. Conclusion

This study supports the evidence that *Bacillus* probiotic supplementation may represent a viable strategy to enhance gonad growth, survival, and coloration in *S. purpuratus* using an entirely plant‐based diet. Probiotic treatments achieved commercial‐grade GSI values (>12%) and approximately two‐fold greater growth rates compared to the same diet without probiotics. Beyond growth performance, *Bacillus* supplementation appeared to enhance urchin survival and gonad coloration potentially through ANFs degradation and β‐carotene conversion to putative 9CEC, outcomes that were also absent in the nonprobiotic control group. Moreover, these findings provide evidence that *Bacillus*‐supplemented plant‐based diets are potentially viable and could offer sustainable alternatives for sea urchin ranching. This biotechnology innovation may offer comparable or superior performance to traditional macroalgae‐ and fishmeal‐based formulations while addressing environmental and economic concerns associated with wild macroalgae harvesting and fishmeal production. If reproducible at larger scales, this approach may provide a practical framework for expanding sea urchin ranching operations while contributing to kelp forest restoration efforts. Future research should examine the relationship between *Bacillus* inclusion levels in aquafeed and the relationship with sea urchin gonad quality parameters—beyond growth and coloration. Additionally, future work should evaluate digestibility performance assays, histopathological assessment of ANF‐induced intestinal damage, profiling of *Bacillus*‐mediated microbiome modulation, and the expression of related genes in *S. purpuratus*.

## Author Contributions

Alfonso Rodríguez completed the experiment, laboratory analyses, statistical analyses, visualization, and wrote the original manuscript. Jeremie Bauer obtained experimental organisms, completed the experiment, visualization, and writing and reviewing. Manuel Acosta and Carmen Paniagua‐Chávez provided equipment and facilities, and writing and reviewing. Jorge Olmos conceptualized and supervised the study, provided funding equipment and facilities, revised the original manuscript, and writing and reviewing.

## Funding

Partial financial support was received from the Secretaría de Ciencia, Humanidades, Tecnología e Innovación (SECIHTI) to Alfonso Rodríguez (CVU 1255009) and Jeremie Bauer (CVU 860611).

## Disclosure

All figures and tables in this manuscript are original and created by the authors or properly cited if adapted from other sources. No permissions were required for the reproduction of material from other sources.

## Ethics Statement

The animal study protocol was approved by the Institutional Review Board of Comité de Bioética del CICESE (Bioethics Committee of CICESE ORGA_ACUA_2025_03 on July 07, 2025).

## Consent

This study did not involve human subjects or patients.

## Conflicts of Interest

The authors declare no conflicts of interest.

## Data Availability

The original contributions presented in this study are included in the article material. Further inquiries can be directed to the corresponding author.
